# A rapid and non-invasive method for measuring the peak positive pressure of HIFU fields by ﻿a﻿ laser beam

**DOI:** 10.1038/s41598-017-00892-4

**Published:** 2017-04-12

**Authors:** Hua Wang, Deping Zeng, Ziguang Chen, Zengtao Yang

**Affiliations:** 1 College of Biomedical Engineering, State Key Laboratory of Ultrasound Engineering in Medicine Co-founded by Chongqing and MOST, Chongqing Municipal Key Laboratory of Ultrasound Engineering in Medicine, Chongqing Medical University, Chongqing, 400016 China; 2 Mechanical and Materials Engineering, University of Nebraska–Lincoln, Lincoln, NE 68588 United States

## Abstract

Based on the acousto-optic interaction, we propose a laser deflection method for rapidly, non-invasively and quantitatively measuring the peak positive pressure of HIFU fields. In the characterization of HIFU fields, the effect of nonlinear propagation is considered. The relation between the laser deflection length and the peak positive pressure is derived. Then the laser deflection method is assessed by comparing it with the hydrophone method. The experimental results show that the peak positive pressure measured by laser deflection method is little higher than that obtained by the hydrophone, confirming that they are in reasonable agreement. Considering that the peak pressure measured by hydrophones is always underestimated, the laser deflection method is assumed to be more accurate than the hydrophone method due to the absence of the errors in hydrophone spatial-averaging measurement and the influence of waveform distortion on hydrophone corrections. Moreover, noting that the Lorentz formula still remains applicable to high-pressure environments, the laser deflection method exhibits a great potential for measuring HIFU field under high-pressure amplitude. Additionally, the laser deflection method provides a rapid way for measuring the peak positive pressure, without the scan time, which is required by the hydrophones.

## Introduction

Focused ultrasound (FUS) has been used to produce localized high intensity for various purposes, especially in the field of medical diagnosis and therapy. One of important applications of FUS to real-world problems is the high intensity focused ultrasound (HIFU) therapy^1, 2^, in which ultrasound energy can be focused into a small volume to heat and destroy the target without causing damage to the intervening tissue. The acoustic characterization of HIFU fields is important both for the accurate prediction of FUS induced bioeffects in tissues and for the development of standards to ensure the safety and efficacy of treatments. Amount all of the acoustic characterization, accurate measurement of the peak positive pressure in HIFU fields is important for predicting thermal effects during HIFU treatments^3–5^. However, the peak positive pressure at focus spot for modern HIFU devices operating at clinical output levels can be up to 10^7^ Pa or higher, and the size of the focal spot is only millimeter or even submillimeter^6, 7^. The high pressures and tight focusing of HIFU devices make accurate acoustic field measurements challenging^3, 4^.

Currently available acoustic pressure sensors used for measuring the ultrasound fields fall into two categories: piezoelectric hydrophones and optical hydrophones^5, 8^, which respond to the pressure averaged over its active sensors. In the pratical applications, the polyvinylidene fluoride (PVDF) membrane hydrophone and fiber optic probe hydrophone (FOPH) are often used for mapping the acoustic fields of a HIFU transducer^9^. Compared with the standard PVDF hydrophones, FOPH systems generally possess small probe size, larger bandwidths, enhanced spatial resolution and reduced directionality, though they are limited by significantly lower sensitivities^10, 11^. However, both of PVDF membrane hydrophone and FOPH have inherent limitations in materials and the procedures. Firstly, the two types of hydrophones generally cannot endure the extreme conditions in the focus of a HIFU field because they are easily damaged by cavitation or thermal effects. To avoid cavitation damage, measurements are typically carried out first at a low output level, and then the pressure amplitudes and distribution at high output levels are extrapolated based on linear propagation model^3, 5, 9^. However, the nonlinear behavior for HIFU devices operating at clinically relevant high powers significantly alters the size and shape of the focal point^12, 13^. Thus the linear extrapolation method cannot provide accurate and satisfactory results. Secondly, due to the hydrophones respond to the pressure averaged over its active sensors, errors in spatial-peak pressure measurement are inevitable^14, 15^. Moreover, the acoustic waveforms measured using broadband hydrophones are strongly distorted due to finite amplitude propagation in the water path. Models for characterizing the magnitude of the spatial-averaging correction are no longer valid and may be significantly in error, leading to peak-pressure underestimates of 100% or more^15, 16^. Thus the hydrophones mentioned above are incapable of accurately determining the true peak positive pressure.

Alternatively, optical methods, which can observe the sound field non-invasively, present a promising way to measure the peak positive pressure of HIFU fields. It is well known that the velocity and frequency of light in water are much higher than those of sound in water. The rapidly changing information in HIFU fields could be extracted by optical manners easily. Currently, optical methods based on Schlieren imaging^17^ and optical diffraction tomography^18^ have been used to quantify the pressure field of ultrasound transducers. Yet, they are limited to the conditions under low-pressure amplitudes associated with linear wave propagation^9^. Moreover, they are difficult to calibrate and some of them just provide time intensive scanning, therefore, having not been a suitable method for quantitative measurements.

Other than the two optical methods introduced above, the light deflection method, based on the optical interference phenomena produced by the periodicity of the refractive index in a sound field, can be utilized to measure the peak positive pressure of HIFU fields non-invasively. Confirmed by Lucas and Biquard^19^, light deflects by ultrasonic waves when the wavelength of sound is larger than optical beam width. This phenomenon was theoretically and experimentally investigated by Kolb *et al*.^20^ and Hargrove *et al*.^21^. Recently, laser beam deflection was utilized to measure the intensity or sound pressure of a focused ultrasound^22, 23^. Butterworth and Shaw^22^ in national physical laboratory (NPL) of UK found that the increasing pressure gradients within the field can induce the expected increase in refractive index gradient and consequential refraction. However, the theoretical analysis of the relation between laser point displacement on the screen and acoustic pressure of the focus was not conducted, and the measurements were made under very low pressure amplitudes, in which the nonlinear acoustic behavior was not involved.

Apart from the methods mentioned above, a novel method, which works non-invasively by monitoring the particle displacement in a HIFU field using acoustic streaming, is presented in ref. 24. However, the method is only applicable to moderate intensity regimes.

In this study, we use a laser beam method for rapidly and quantitatively measuring the peak positive pressure of HIFU fields. Based on the light deflection caused by acousto-optic interaction, the relation between laser point displacement on the screen and peak positive pressure of HIFU fields is analyzed theoretically and experimentally. The paper is organized as follows. Firstly, experimental results are presented. A comparison between the laser deflection results and hydrophone results is described. Secondly, some limits on the laser deflection method are discussed. Finally, the method part is presented. In this part, the theory of laser beam deflection in ultrasound field is introduced. The experimental setup is demonstrated.

## Results

**Figure 1:**
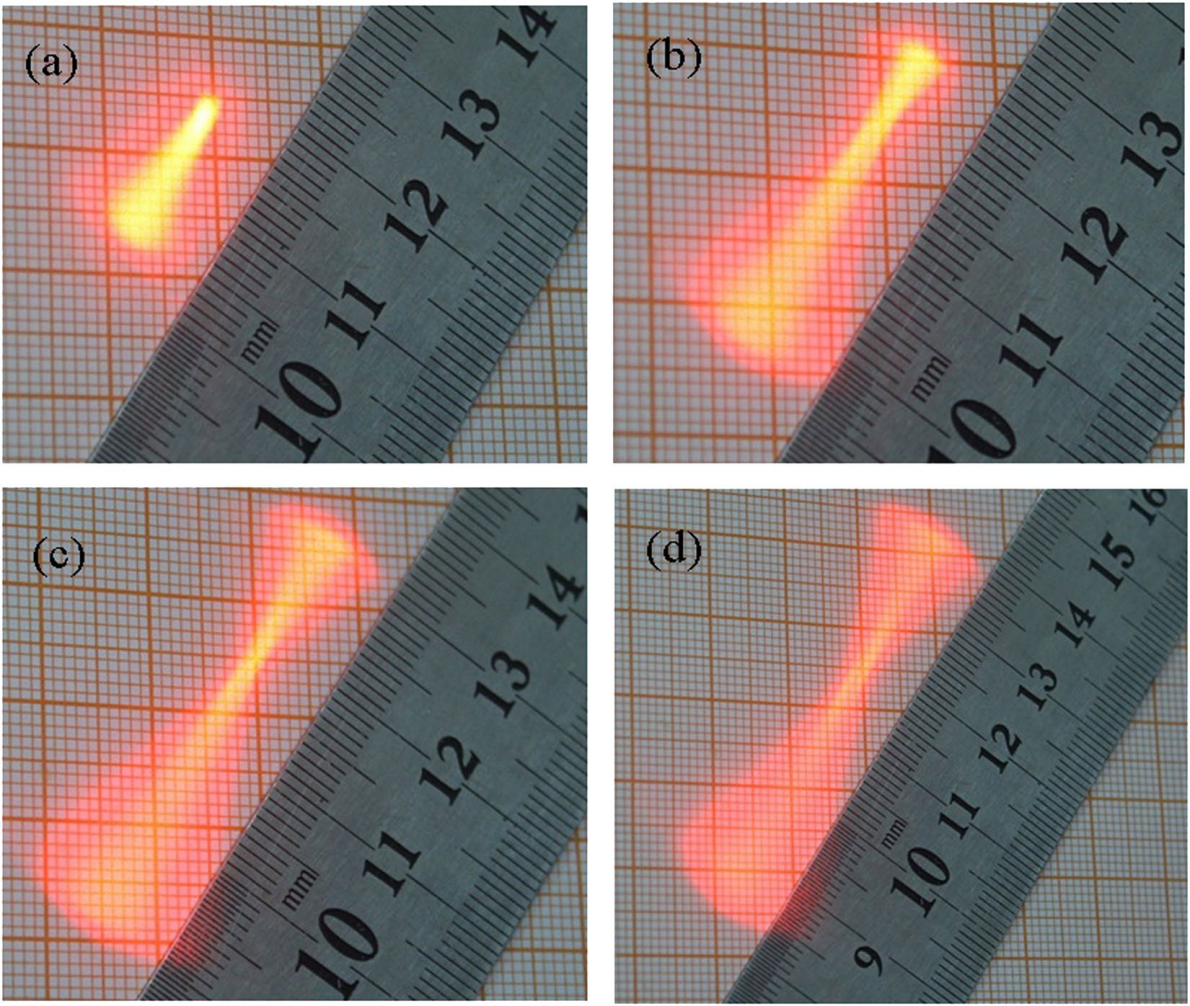
Time-averaged laser spots projected on the screen with varying input voltage to the transducer. (**a**) The input voltage to the transducer is 30 V. (**b**) The input voltage to the transducer is 70 V. (**c**) The input voltage to the transducer is 110 V. (**d**) The input voltage to the transducer is 150 V.

To verify the theory we present, experiments was setup and made at 17 °C. The deflection length of laser spot projected on the screen was measured. Consider that the theory we present is not valid when the cavitation occurs. In the experiments, the running time for the transducer was set short enough in order to prevent the occurrence of cavitation in the focus region. Figure 1 shows the time-averaged deflection length of the laser beam with varying input voltages applied to the transducer. Note that the focal intensity increases with an increase in the input voltage applied to the transducer. It is seen that the deflection length increases with the increase of ultrasonic intensity, indicating that the increasing pressure gradients within the field are causing the expected increase in refractive index gradient and consequential increased deflection. The result agrees well with previous findings^22^.

**Figure 2:**
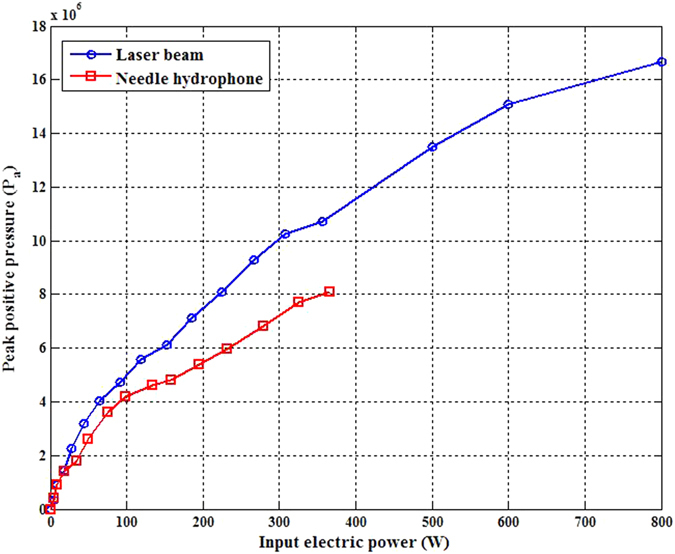
The peak positive pressure with varying input electric power of the transducer.

To assess the accuracy of the present method, a needle hydrophone (HNA-0400, Onda Corp., Sunnyvale, USA) was utilized for measuring the peak positive pressure of the HIFU field under low amplitude conditions. As shown in Fig. 2, an experimental comparison of two methods for measuring the peak positive pressure with varying input electric power of the transducer is presented. The results in Fig. 2 show that the peak positive pressure at low output levels measured by laser beam is in good agreement with that obtained by the hydrophone. However, the true peak positive pressure at the focus was not acquirable in our experiments because the hydrophone would be easily damaged at higher source levels. It is interesting to note that the laser deflection method still remains in operation at high output levels. In our experiments, the highest pressure measured by the laser beam was 1.67 × 10^7^ Pa, much higher than that obtained by the hydrophone. Though this method had ability to detect even higher pressure, the increase in power input to the transducer was terminated to avoid transducer damage. Noting that the Lorentz formula remains applicable to high-pressure environments, the laser deflection method has a great potential for measuring HIFU fields under high-pressure amplitude, which is also confirmed experimentally in Fig. 2.

Additionally, Fig. 2 illustrates that the peak positive pressure measured by laser beam is little higher than that obtained by the hydrophone. Considering that the errors in spatial-peak pressure measurements and the influence of waveform distortion on hydrophone spatial-averaging corrections, the peak positive pressure at the focus measured by the hydrophones is always underestimated^15, 16, 25^. We conclude that the results obtained by the laser deflection method are in reasonable agreement with that obtained by the hydrophone method. Moreover, the laser deflection method is assumed to be more accurate than the hydrophone method due to the absence of the errors in spatial-averaging measurement and the influence of waveform distortion on hydrophone corrections.

In our experiment, the proposed laser beam width was comparable to the focal region size of the HIFU field, which ensured that there definitely exists one ray passing through the point of peak positive pressure. Once the projection on the screen was formed, the peak positive pressure of the HIFU field was determined by Eq. (10) immediately. Compared with hydrophone measurement, there is no need to scan the whole acoustic field to obtain the peak positive pressure. Thus the laser deflection method can provide a rapid way to measure the peak positive pressure of HIFU fields. And the effects which could influent the accuracy of the hydrophone measurement^15^ can be avoid by laser deflection method.

## Discussion

The Food and Drug Administration in the US and other regulatory agencies have required waveform measurements in water as part of the approval process for ultrasound medical devices. Specific protocols have been described for diagnostic ultrasound and shock wave lithotripsy, but not yet for HIFU. Accurate measurement of the peak positive pressure is critical for predicting thermal effects during HIFU treatments. Though the peak positive pressure of HIFU field can be determined, the pressure distribution of whole HIFU field is not required in this study. Thus the spatial resolution of pressure field for this method is limited. Though the temporal information for the waves at the point of maximum can be acquired theoretically using our method presented, the technical difficulty in realizing the conditions for light deflection at ultrasonic frequencies is not yet overcome.

Note that the nonlinear model for analyzing the HIFU field is necessary. In our study, only the second-harmonic generation is used to characterize the HIFU field. If higher order solution for modeling HIFU field is introduced, the measured result by the proposed method would be much closer to the true pressure amplitude of HIFU field. However, the analytical expression to consider all the nonlinear phenomena is hard to be realized.

This study presents a non-invasive way for measuring the peak positive pressure of HIFU field. Moreover, this method would be used in other fields for measuring the high pressure, such as shock waves pressure, and that is beyond the scope of this paper.

## Methods

### Theory

Optical refraction produced by sound waves is a useful tool for the study of sound field. Lucas and Biquard^19^ firstly found that light refraction occurred when the width of the light beam is much smaller than the wavelength of sound. In this paper, we wish to describe a new application of this effect to rapidly and quantitatively measure the peak positive pressure of HIFU fields.

### Theory of laser beam deflection in ultrasound field

**Figure 3:**
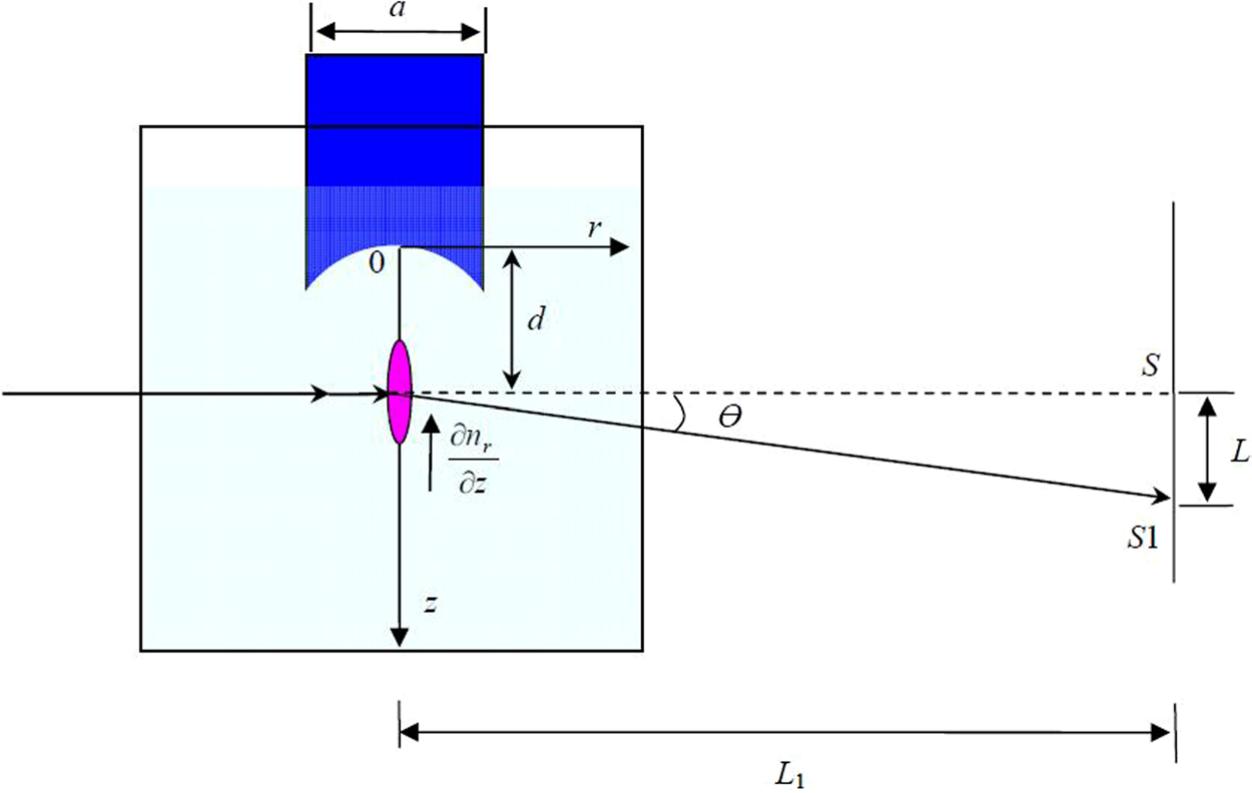
Schematic view of light deflection by ultrasound.

This method makes use of the refraction of light in a liquid which has a refractive index *n*_*r*_ varying sharply in the focal spot of a HIFU field, both in horizontal direction and vertical direction. As shown in Fig. 3, a light beam traverses the focal region of the HIFU field in a horizontal direction, therefore normal to the direction of the gradient of the refractive index *n*_*r*_. Then the deflected laser point is projected perpendicularly on a screen at the front of a water tank. In passing through a layer of liquid, the beam is deflected from its original direction due to the spatial gradient of the refractive index induced by ultrasonic waves.

The density of water varies when acoustic waves transmit in water, and the medium refractive index varies with the change of density. According to the Lorentz formula^26^, the variation in pressure produces a variation in the refractive index. The Lorentz formula gives for Δ*n*

1 $${\rm{\Delta }}n=K{\rm{\Delta }}p$$

where Δ*n* is the variation of refraction index, Δ*p* is the variation of acoustic pressure, and *K* is the piezo-optic coefficient which describes the variation of the refractive index. Due to the strong acoustic nonlinearity in HIFU fields, the pressure of high order terms should be considered. However, as the value of the piezo-optic coefficient is tiny^27^, the high-order variation in the refractive index caused by optical nonlinearity can be ignored in equation (1). It should be pointed out that the influence of cavitation on the Lorentz formula is strong. When cavitation occurs, the medium in the focal region is discontinuous and the relation of the variation of refraction index Δ*n* to the variation of acoustic pressure Δ*p* is uncertain. Thus, equation (1) is not valid when cavitation occurs.

The point of incidence projected on the screen is displaced from *S* to *S*_1_. Let we denote the length of *SS*_1_ as *L*. The deflection angle *dθ*_*r*_ for the point on *r*-axis is given as

2 $$d{\theta }_{r}=\frac{1}{{n}_{0}}\frac{d{n}_{r}}{dz}dr$$

where *n*_0_ is the refractive index of water (*n*_0_ = 1.33) in the absence of ultrasound.

The total deflection angle *θ* of the light beam passing through the liquid of an effective length *L*_*e*_ can be derived as

3 $$\theta ={\int }_{{L}_{e}}d{\theta }_{r}=\frac{1}{{n}_{0}}{\int }_{-\frac{{L}_{e}}{2}}^{\frac{{L}_{e}}{2}}\frac{d{n}_{r}}{dz}dr$$

For a HIFU field, the effective length *L*_*e*_ approximately equals to the focal size in the horizontal direction. Noting that the piezo-optic coefficient *K* is of the order of 10^−10^ Pa^−1^, the total deflection angle *θ* caused by a tiny variation of refraction index in HIFU fields is very small. Then, the light deflection length *L* projected on the screen is obtained as

4 $$L={L}_{1}\,\tan \,\theta \approx {L}_{1}\theta $$

where *L*_1_ is the optical path from the middle of the transducer to the screen. In the experiment, *L*_1_ is always designed to be a few meters to ensure that the projection on the screen is longer than the laser beam width to permit precise measurement.

**Figure 4:**
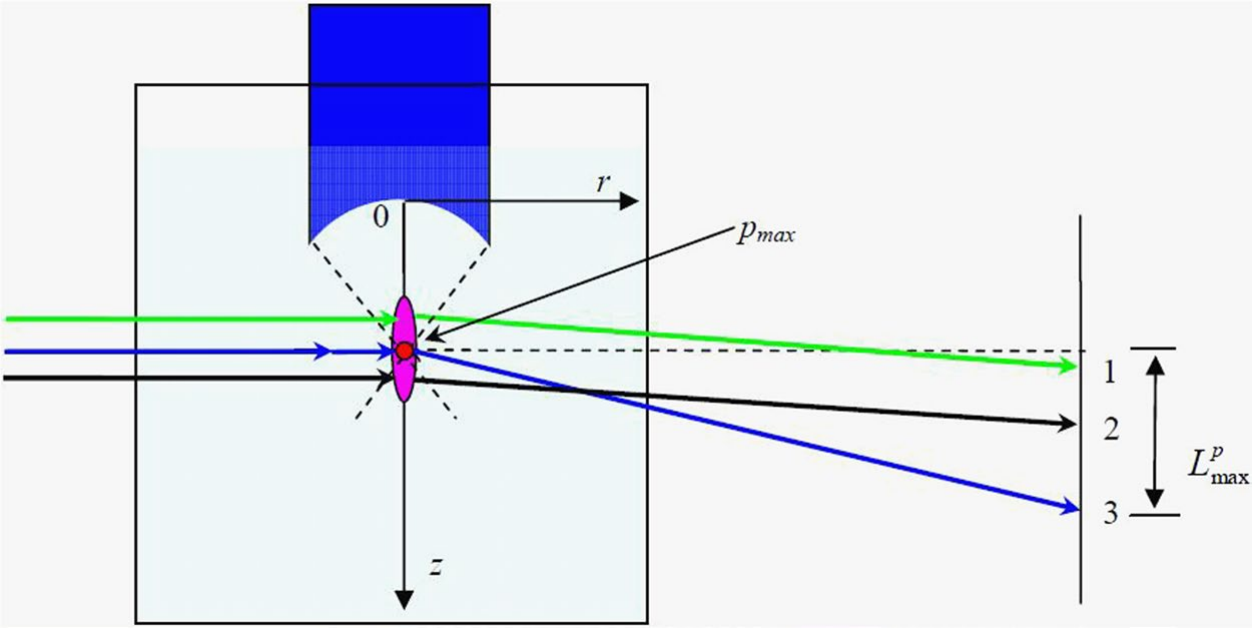
Schematic diagram of the largest deflection length projected on the screen.

In order to realize a rapid measurement of the peak positive pressure of HIFU fields, the width of such laser beam we proposed is comparable to the focal size of HIFU fields. Note that the laser beam which serves as the light source is composed of countless rays according to the geometrical optics theory. The width of each ray is much smaller than the wavelength of sound, satisfying the conditions of light refraction^19^. As shown in Fig. 4, in order to ensure that there definitely exists one ray passing through the point of peak positive pressure in the focal region, the laser beam of a width comparable to the focal size of the HIFU field is proposed. As demonstrated in Fig. 4, the ray no. 3 which passes through the point of peak positive pressure has the largest deflection length on the screen according to Eq. (4). Thus we can determine the peak positive pressure by measuring the largest deflection length on the screen. Compared with hydrophone method, the laser deflection method can determine the peak positive pressure of HIFU fields immediately by the largest deflection length on the screen, without the scan time, which is required by the hydrophone measurement.

### Laser beam deflection in the focused ultrasound field with nonlinear propagation

In the theoretical analysis of a focused ultrasonic source, it is necessary to consider the nonlinear phenomena such as harmonic generation, waveform distortion, and shock formation. Owing to the complexity of nonlinear phenomena in focused sound beams, analytical investigation to consider all the nonlinear phenomena is hard to be realized. In this paper, we begin by restricting our attention to second-harmonic generation by a sound beam radiated at a single angular frequency ω. The solution describing the sound-pressure waveform *p* is in terms of the following expansion^28^

5 $$p(r,z,t)={p}_{1}(r,z,t)+{p}_{2}(r,z,t)$$

where *p*_1_ is the linear solution of KZK parabolic wave equation^29^ for the pressure at frequency *ω*, and *p*_2_ is a small correction to *p*_1_.

6 $${p}_{n}(r,z,t)=\frac{1}{2j}{q}_{n}(r,z)\exp [jn(\omega t-kz)]+{\rm{c}}.{\rm{c}}.,$$

in Eq. (5), *q*_1_ and *q*_2_ are complex pressure amplitudes, and c.c. denotes the complex conjugate of preceding terms.

To characterize the focused ultrasound field, source of Gaussian amplitude shading are frequently used. Consider the lossless solutions associated with second-harmonic generation in Gaussian beam. The *q*_1_ and *q*_2_ can be expressed as:

7 $$\begin{array}{rcl}{q}_{1}(r,z) & = & \frac{{p}_{0}}{1-(1+j{G}^{-1})z/d}\exp [-\frac{(1-jG){(r/a)}^{2}}{1-(1+j{G}^{-1})z/d}],\\ {q}_{2}(r,z) & = & \frac{j{P}_{2}}{(1-jG)}\frac{\mathrm{ln}[1-(1+j{G}^{-1})z/d]}{1-(1+j{G}^{-1})z/d}\exp [-\frac{2(1-jG){(r/a)}^{2}}{1-(1+j{G}^{-1})z/d}].\end{array}$$

where *G* = *z*_0_/*d* = *ka* ^2^/2*d*, and ${P}_{2}=\beta {p}_{0}^{2}{k}^{2}{a}^{2}/(4{\rho }_{0}{c}_{0}^{2})$.

Noting that *p* is a slowly varying function of *z* at high frequency near the focal region, we assume $\frac{\partial {q}_{i}}{\partial z}\ll k{q}_{i}$ ^28^. And thus we have

8 $$\frac{\partial {p}_{n}(r,z,t)}{\partial z}\cong -jnk\frac{1}{2j}{q}_{n}(r,z)\exp [jn(\omega t-kz)]+{\rm{c}}.{\rm{c}}.,$$

When the light passes through the focus point (*z* = *d*), substituting of Eqs (1) and (8) into Eq. (4), the light deflection length can be obtained as

9 $$\begin{array}{rcl}L & = & {L}_{1}\frac{K}{{n}_{0}}\frac{1}{2j}\frac{1}{{G}^{-1}}k\sqrt{\frac{-j{G}^{-1}{a}^{2}}{(1-jG)}\pi }\{[{p}_{0}\exp [j(\omega t-kd)\\  &  & +j{P}_{2}\frac{\sqrt{2}\,\mathrm{ln}(-j{G}^{-1})}{(1-jG)}\exp [j2(\omega t-kd)]\}+{\rm{c}}.{\rm{c}}.\end{array}$$

where c.c. denotes the complex conjugate of preceding terms.

The Eq. (9) contents the temporal distribution information of light deflection. In each period, two peaks of deflection, the peak positive deflection length ${L}_{{\rm{\max }}}^{p}$ and the peak negative deflection length ${L}_{{\rm{\max }}}^{n}$, can be determined by equation (9). Then, the largest deflection length for the laser beam projected on the screen is given as

10 $${L}_{{\rm{\max }}}={L}_{{\rm{\max }}}^{p}+{L}_{{\rm{\max }}}^{n}$$

### Experimental setup

**Figure 5:**
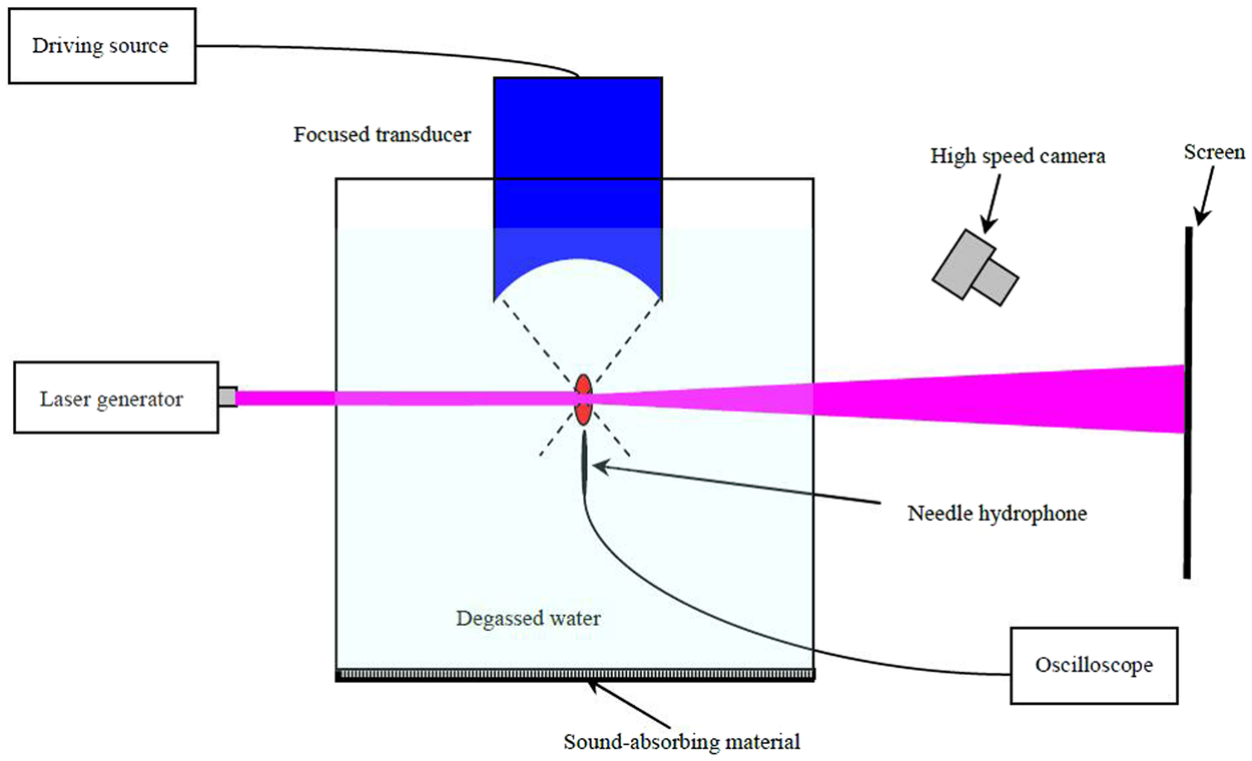
Schematic diagram of experimental setup.

Experimental setup is shown in Fig. 5 (with a similar setup to that shown in Fig. 3). All measurements were taken in degassed water to avoid the cavitation. For 17 °C water, the piezo-optic coefficient *K* = 1.503 × 10^−10^ Pa^−1^. As shown in Fig. 5, a laser beam with a width of 4 mm was generated by a laser generator (HN-1200, Peking University Factory of Physics Department, Beijing, China). The width of the laser beam was comparable to the size of focal spot. A screen was placed at the front of a water tank, where the laser point was projected perpendicularly from the rear of the tank through the focal region of a HIFU transducer, first without the presence of the field, then with increasing focal intensity. The optical path *L*_1_ of length 4.7 m was used. A digital camera (EOS 350D, Canon, Tokyo, Japan) was used to record the length of the laser deflection on the screen. The ultrasonic transducer (Chongqing Haifu Medical Technology Co., Chongqing, China) which was submerged in the tank had a fundamental frequency of 0.83 MHz, a radius of curvature of 50 mm and an aperture of 50 mm. The transducer was powered by an ultrasonic driving source (AG1024, T&C Power Conversions Inc., New York, USA) in continuous-wave mode. A commercial needle hydrophone (HNA-0400, Onda Corp., Sunnyvale, USA) was employed to measure the peak positive pressure of focused acoustic field. An oscilloscope (Agilent DSO7014B, Agilent Technologies, Santa Clara, USA) was used to record the peak-to-peak voltages of the needle hydrophone.
